# Stay with your community: Bridges between clusters trigger expansion of COVID-19

**DOI:** 10.1371/journal.pone.0242766

**Published:** 2020-12-03

**Authors:** Yukio Ohsawa, Masaharu Tsubokura

**Affiliations:** 1 Department of Systems Innovation, School of Engineering, The University of Tokyo, Tokyo, Japan; 2 Department of Radiation Health Management, Fukushima Medical University School of Medicine, Fukushima, Japan; Rutgers The State University of New Jersey, UNITED STATES

## Abstract

In this study, the spread of virus infection was simulated using artificial human networks. Here, real-space urban life was modeled as a modified scale-free network with constraints. To date, the scale-free network has been adopted for modeling online communities in several studies. However, in the present study, it has been modified to represent the social behaviors of people where the generated communities are restricted and reflect spatiotemporal constraints in real life. Furthermore, the networks have been extended by introducing multiple cliques in the initial step of network construction and enabling people to contact hidden (zero-degree) as well as popular (large-degree) people. Consequently, four findings and a policy proposal were obtained. First, “second waves” were observed in some cases of the simulations even without external influence or constraints on people’s social contacts or the releasing of the constraints. These waves tend to be lower than the first wave and occur in “fresh” clusters, that is, via the infection of people who are connected in the network but have not been infected previously. This implies that the bridge between infected and fresh clusters may trigger a new spread of the virus. Second, if the network changes its structure on the way of infection spread or after its suppression, a second wave larger than the first can occur. Third, the peak height in the time series of the number of infected cases depends on the difference between the upper bound of the number of people each member actually meets and the number of people they choose to meet during the period of infection spread. This tendency is observed for the two kinds of artificial networks introduced here and implies the impact of bridges between communities on the virus spreading. Fourth, the release of a previously imposed constraint may trigger a second wave higher than the peak of the time series without introducing any constraint so far previously, if the release is introduced at a time close to the peak. Thus, overall, both the government and individuals should be careful in returning to society where people enjoy free inter-community contact.

## Introduction

The spread of infection during a pandemic is a major public health challenge. Therefore, its prediction and ensuring that appropriate precautions are taken are the critical components in determining an efficient/effective solution. The novel coronavirus (SARS-CoV-2), which was first reported in Wuhan, Hubei Province, China in December 2019, spread rapidly throughout the world, thus causing the COVID-19 disease. This disease was declared a pandemic relatively quickly by the World Health Organization (WHO) and, as of October 2020, over 1.2 million deaths had occurred worldwide and more than 46 million cases have been confirmed. In this context, various measures for the prevention of infection, mostly following the WHO recommendations, have been presented by the authorities in each country. In addition to personal preventive measures such as handwashing and wearing face masks, social preventive measures such as physical distancing and drastically restricted individual movements (lockdowns) are being implemented. Such preventive measures are implemented by several authorities, with varying degrees of success. However, questions such as “will the spread of the disease be suppressed as expected when people distance themselves from others?” and “who should we avoid contact with?” are often asked by people who are not very knowledgeable in this regard.

To respond to the aforementioned questions and obtain effective prevention measures, we herein present a model for simulating the spread of virus infection in a social network of people considering an urban environment. We utilized the social network model, instead of the population models with differential equations, such as the SIR models, because our aim is to clarify the consequences of the actions of people that either generate, prune, or perturb the local contact within neighbors, and accordingly, to derive the knowledge required for determining appropriate measures that need to be imposed for preventing the spread of infection. This advantage of the network-based model has been highlighted by Karaivanov in [1], wherein, its differences from population-based models such as SIR [2] and its extensions [3–7] were clarified. Here, the effects of preventive actions such as lockdown, its release, and of infections via edges bridging far-apart nodes, on infection spread have been evaluated.

Various network models of infection have been built to predict its spread and to ensure informed decision making concerning its control and prevention [8–12]. In particular, Vazquez [8] modeled the dynamics to explain the increase of infections in the early stage based on the power-law followed by exponential suppression. This model has been utilized and extended, firstly, to obtain an indicator of the failure of the current containment efforts and the beginning of the end of the pandemic [9], and secondly, to compare the temporal trends of daily cases in various countries [10]. Strategies for network manipulation have also been explored, one of which is the control of the dynamics of disease spread via interaction with the features of a disease network, such as the infection rate [11]. Another strategy is network optimization, which involves removing/rewiring links that represent an NP-hard problem [12]. The solutions for these problems include the top-down approach, for example, to suppress infection rates as well as the bottom-up approach, which enables individuals to recognize their critical in-network contacts. Therefore, the factors/information that are/is critical when designing policies for controlling the epidemic spread can be obtained by evaluating the network dynamics. The guidelines to adhere to and information obtained from such research could be beneficial for designing policies to control epidemics in a timely manner.

There exists a considerable amount of knowledge related to the general theories that are applicable to various propagation phenomena in social networks. For example, the cascades in skewed human networks are triggered by large degree nodes, whereas the small degree nodes may trigger cascades in other networks [13]. Kurahashi and Saito [14] simulated the diffusion of innovation in artificial social networks, resulting in delayed propagation from high-degree nodes to low-degree nodes, which has a greater impact if informative and nominal contacts from neighboring nodes are mixed. They discussed the mutual influence of high-degree nodes on low-degree nodes, with the latter as the main focus. Ziff and Ziff [9], Kurahashi and Saito [14], and other researchers working on network dynamics, have contributed valuable information to help determine the optimal intervention strategies [15].

In our network model representing human society, we assumed that people are eager to attend places where several people gather. This tendency may exist not only because of the other people present in those places but also because the site may be a particular attraction, either abstract or real. Furthermore, in such a place, a new person may come into close contact with other people, touch objects, and breathe the air, which are similar elements to those of the social dynamics of scale-free networks (SFNs) where the degree distribution follows the power law. This distribution has been explained by the theory of network generation in which new nodes become connected to high-degree nodes, as in the later section of this paper [16]. However, the following real-space and real-time constraints on the dynamics exist: 1) the restriction on the amount of time a person has to be able to meet other people, 2) the restriction of space, and 3) the interest in meeting other people. Considering 1 and 2, we assume that the number of people each person actually meets is restricted due to the constraints on the available time and space for the meeting. Accordingly, we introduced *W*, defined as the upper bound on the number of people that each person actually meets. And, to represent the 3^rd^ constraint, we introduced *m*_0_, which indicates the number of other people each person chooses to meet either singly (one by one) or in a group.

In this study, we aim to find evidence to further the discussion using simple simulations involving two types of constrained SFNs. From the simulated cases with varying values of *W* and *m*_0_, we ascertained the effects of the bridges between communities on infection spreading in a manner that is intuitively understandable by anyone who desires to obtain a preventive measure for practical application.

## Materials and methods

### Hypothesis

**Fig 1:**
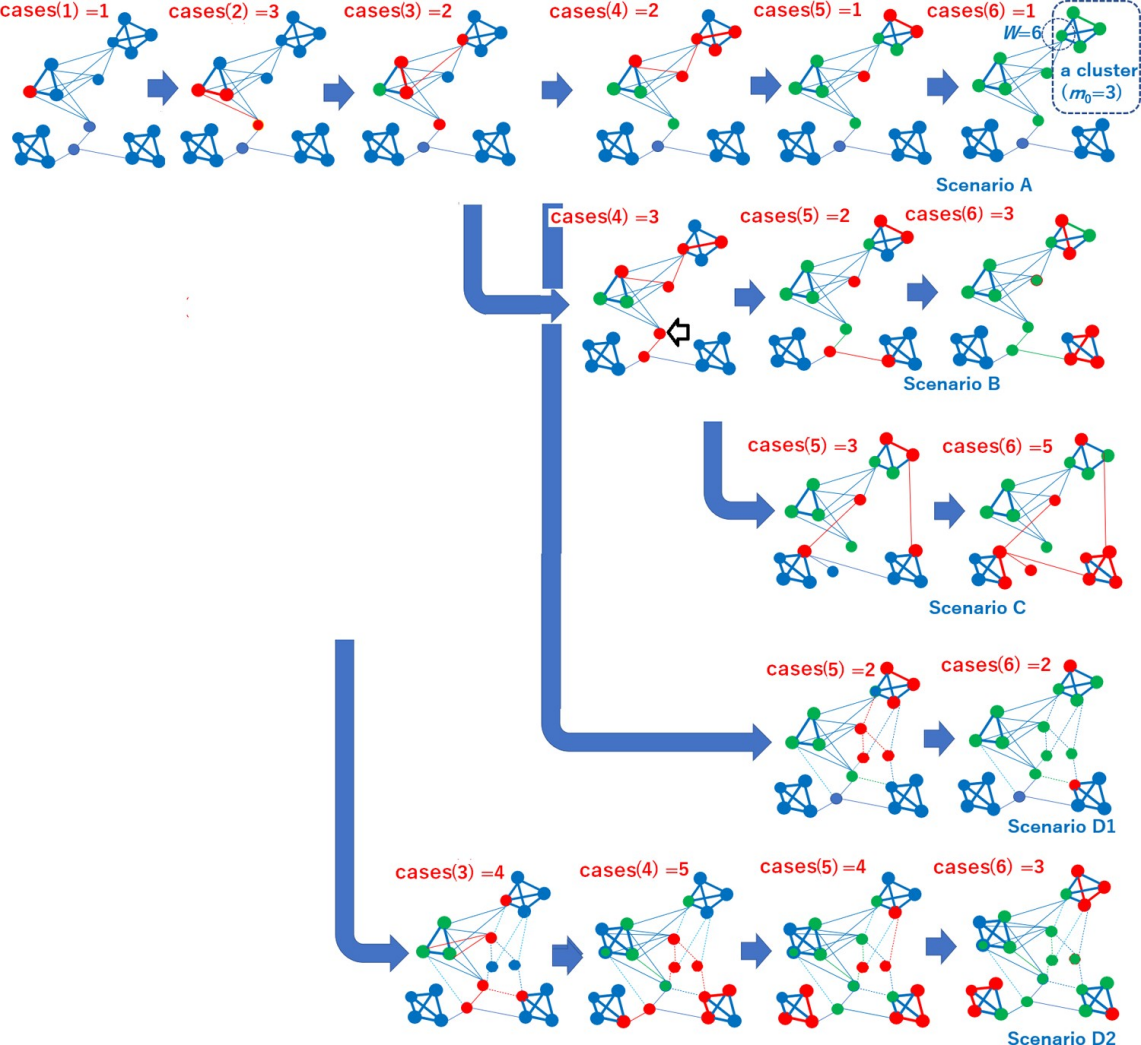
Four scenarios of infection spreading in a social network. The value of cases(*t*) indicates the number of new infection cases at time *t* e.g., the *t*-th week, in each scenario. This figure presents a simplified conceptual graph. For example, the clusters generated initially should be smaller than those generated later in Algorithms 2 and 3.

Each image in Fig 1 indicates a social network. Each node represents a person, a place, or an object in that place. Each edge represents the possibility that the entities represented by the two nodes can interact. Moreover, each cluster with thick solid edges depicts a group of people who choose to meet each other. The people in such a group are mutually connected via strong ties, whereas the thin lines represent weaker ties bridging the groups via personal interests in external activities. Here, the red nodes represent people who are chosen randomly from the neighbors of infective nodes in the preceding step with a certain probability (*p*_active_ defined later) and have currently acquired infectivity, i.e., the ability to infect others. In this figure, the delay between acquiring the infection and becoming infected is ignored. The blue nodes are not yet infected, and the green nodes represent people who have lost their infectivity to others. In this study, we set the following four hypotheses based on networked models of society, as illustrated in Fig 1.

*Hypothesis 1*: The height of the peaks of the number of infection cases in the time sequence depends on the upper bound of the number of people each member actually meets (*W*), and the curve of this dependency varies with the number of people they choose to meet (*m*_0_).

*Hypothesis 2*: New waves, such as a second wave, of infection spread may occur without external events such as an increase in in-bound travelers or a release of governmental constraints on social interaction.

*Hypothesis 3*: The change in the social structure, which happens when each person changes the choice of neighbors, i.e., whom to meet, can trigger a wider spread of infection.

*Hypothesis 4*: The release of the constraints on social interaction can cause a wide spread of infection if it is introduced too soon.

We first considered Hypothesis 1, and evaluated the other hypotheses subsequently on the simulated peak heights considered in this study because the peak height indicates the load on hospitals and other social facilities that deal with infected patients at a certain time. Hypothesis 1 can be comprehended by viewing Scenario A, illustrated in Fig 1. As shown in the top right of the figure, *W* corresponds to all the edges connected to a member, and *m*_0_+1 corresponds to the number of thick solid edges in a cluster (as shown, *m*_0_ for a cluster is obtained in the initial step in Algorithms 2 and 3 described later). A cluster can be regarded as a community of people. The value of *W*–*m*_0_ represents the width of the bridge between communities that corresponds to the number of thin edges between clusters and contributes to enabling virus infections to spread from one community to the others. For a larger *W*–*m*_0_, the peak of infection cases is expected to increase because the infection spreads widely via inter-community bridges.

Hypothesis 2 corresponds to Scenario B illustrated in Fig 1. Here, due to a small extension of the reproductive period of the arrowed node, the virus infects new communities. Consequently, people (nodes) in a new community become infected, which results in the appearance of a new wave. Hypothesis 3, which corresponds to Scenario C here, considers the movement of edges to connect infected people with infectivity and the non-infected people who have not yet acquired immunity. Hypothesis 4 corresponds to Scenarios D1 and D2, wherein the restriction on the interaction of people is released, thereby causing the edges to emerge. If a longer time has passed since the peak of the infection spread until the time of release, as in Scenario D1, the expansion will be less severe than in Scenario D2 because a certain percentage of people in the community have already been infected. On the contrary, in Scenario D2, the restriction is released at a time when fresh clusters, wherein the majority of people have not yet been infected, still exist with links to infected people. As a result, the peak of the number of infected people rises, which exceeds the peak before the release.

### Scale-free network as backbone

An established procedure for generating an SFN [16] is described as follows: Hereafter, *N* indicates the total number of nodes to be included in society. *V* and *E* represent the sets of nodes and edges in the graph, respectively, of which the combination is represented by graph *G*. Graph *G* starts from a clique of *m*_0_ nodes and grows incrementally by adding one node_*i*_ each time. When adding the new node, *m*_0_ nodes are chosen as new link destinations from node_*i*_ according to the probabilities specified in proportion to the node degrees (deg(node_*j*_) for node_*j*_). In other words, the probability that node_*i*_ chooses to be connected to node_*j*_ via a new link is given in Eq (1), and based on this probability, node node_*j*_ is chosen to be a neighbor of node_*i*_ in *G*. This creation of connection continues until *m*_0_ neighbors are chosen for node_*i*_.

$$p_{connected}\left(j\right)=\frac{deg\left(node_{j}\right)}{\sum_{node_{j}\in V}deg\left(node_{j}\right)}$$ (1)

Thus, *m*_0_ is the initial degree of a new node, which is the number of edges connected to a new node. Because node_*j*_ is not chosen deterministically by the ranking of deg(node_*j*_) but probabilistically by *p*_connected_(*j*), the node is chosen using a random function provided in Line 8 of Algorithm 1. That is, the values of deg(node_*j*_), multiplied by a random value between 0 and 1, are compared for ranking the nodes in *G* to select the top *m*_0_ nodes. In real human society, *m*_0_ can be regarded as the number of people that each person intentionally meets, which is all the people that a person can meet separately, or in a group, only if preferred. After the intended meetings, the person may come into contact with other nodes, each of which chooses *m*_0_ other nodes to meet. If the edge called edge_*i*, *j*_ below is included in *E*, node_*i*_ and node_*j*_ are neighbors of each other.

### Algorithm 1: Generation of scale-free network (SFN)

1: *G*: = {*V*, *E*}, *E* = {}

2: *V* = {node_1_,node _2_, …, node _*m*0_}

3: *E* ←*E*+ {edge _*i*, *j*_} **for all** node_*i*_, node_*j*_ **∈** *V*_*k*_ (*i*≠*j*)

4: **for** *i* **=** *m*_0_+1**:** *N* **do**

5:  **add** node_*i*_ **to** *V*

6:  deg(node_*i*_) = *m*_0_

7:  **for** *j* = *m*_0_+1: *N* (*i ≠ j*) **do**

8:    **if $rank_{node_{j}\in V}\text{random}\left(1\right)\;\text{deg}\left({\text{node}}_{j}\right)\leq m_{0}$**

9:      *E* **←** *E +* {edge _*i*, *j*_}

10:       deg(node_*j*_) ← deg(node_*j*_)+1

11:    **end if**

12:  **end for**

13: **end for**

As presented by Barabasi and Albert [16], the distribution of node degrees in the network generated by Algorithm 1 follows the power law, meaning that a few nodes in *V* occupy a large portion of the edges in *E*. In a conventional random graph (Erdős–Rényi's model [17, 18]), where pairs of nodes are connected via edges by a given probability, the degrees of nodes in the network follow the Poisson distribution. On the other hand, in the small-world network (Watts Strogatz model [19]), where a certain percentage p_*replace*_ of edges in a regular graph, wherein all the nodes have an equal degree, are replaced with new edges in which one end is moved to a randomly selected node. Moreover, small-world networks have been used as a model to capture the contacts of people where local communities and their weakly bridging ties contribute to the spread of infection [20, 21]. However, a small-world network self-generates from a regular graph and moves a certain number of randomly selected edges connected from each node, which does not match the behavior of people intending to create a community in the real world. As a matter of degree distribution, in a small-world network, all the nodes take the same degree as in a regular graph for p_*replace*_ = 0 and the degrees follow the Poisson distribution similar to that in a random graph for p_*replace*_ = 1. In this sense, we can say that the power law, which defines the SFN, is a particular characteristic of the network generated by Algorithm 1. Networks, such as the world wide web and online communities [22], cellular networks in biology [23], patients of sexually transmitted diseases [24], and so on, have been known to follow the power-law degree distribution. We also find studies wherein SFN has been utilized as a model for the analysis of infection dynamics [25]. Furthermore, the network of locations, corresponding to rooms in this paper, has been shown to form an SFN [20], and these considered networks model the mixture of people and places. Accordingly, the idea of using an SFN as a backbone is at least partially supported for modeling infection of viruses in the real-space social activities of people. Note that we borrow the process for network generation rather than the degree distribution for the social network simulation in this paper.

Another and more important reason why we choose the SFN generated by Algorithm 1 as the basis of our model is that we adopted the objective to clarify the effect of contact with external communities caused by connections due to unintentionally coming into contact with others such as the thin *W*-*m*_0_ edges in Fig 1. An individual in a real society may not distinguish between a bridge between communities and other relationships but can distinguish a relationship one intentionally made by oneself with others and unintentional contacts made from the side of others. Therefore, in this study, we chose to create a social network model wherein each node (say *X*) chooses *m*_0_ neighbors, thus intentionally following the generation process of SFN above and regard connections from other nodes as unintentional contacts for *X*.

We also compared the original SFN above and its recently proposed extensions such as the mediation-driven attachment model [26, 27], where each new node becomes connected to nodes that are of higher degree and are linked to a larger number of low-degree nodes. We did not choose this model for the present study considering that an attendee in a real-space meeting, while talking to or touching a person, seldom considers whom that person is connected to on other occasions. After all, considering the suitability of each model for the understanding of the social life of people in our scope, we take the network generation in the original SFN by Barabasi and Albert as the backbone and modify it by introducing constraints that correspond to the real-life spatiotemporal restrictions, as presented in the next section.

### Constrained scale-free networks considering spatiotemporal restrictions

The SFN may be, as discussed above, regarded as a useful model for capturing human social behaviors provided that no restrictions are in place with regard to the extent of social interaction of each person. However, the physical constraints in a real-life environment restrict the width of the room where people may be allowed to gather and the time of gathering; a person is, hence, unable to meet as many people as they would ideally like to.In this sense, the original SFN cannot be used in its original form to model the real social behaviors of the people. Therefore, we modified the process as follows: We considered not only people but also rooms, objects, or air in the rooms as nodes at which people gather (similar to people for whom several people gather to meet) because the virus is coated in the saliva of people and emitted to other people via the objects or the air in the meeting rooms. Accordingly, we imposed constraints corresponding to the spatiotemporal restrictions in the two types of artificial networks comprised of people and objects/places.

The first artificial network used in this study is the SFN with spatiotemporal constraints (Algorithm 2: SFN-SC). A feature of the SFN-SC is that it can start from multiple cliques rather than a single clique, which was used in the abovementioned original SFN. This means that we can assume that multiple (*K* >1) groups of people attract other people who arrive later, which is a generalization of the original SFN that corresponds to setting *K* equal to 1. A spatiotemporal constraint is given by the specified upper bound *W* of the degree of the link destination node, which is a partner of connection for the new node added each time, as shown in Line 10 of Algorithm 2. If the destination node represents a room, *W* means the maximum room capacity of people present in it. The *m*_0_ nodes to which this room directs the virus via its *m*_0_ out-going edges, as shown in Lines 10 through 13, may correspond to the facilitators of the meeting. These facilitators direct their outgoing edges to other nodes, which means to inform about (e.g., call for attention or report the achievements of) the meeting to those who are out of the room. If the destination node represents a person, other nodes that arrive later to be connected to that node are the people who choose to meet the person. Because these late-arriving nodes may wish to meet in the same room at the same time, in both cases, where the destination node can be a room or a person, we add Lines 17 through 25 to demonstrate that a group can be formed by the late-arriving nodes, which may infect or get infected from the environment.

### Algorithm 2: SFN with spatiotemporal constraints (SFN-SC)

1: *E* ← {}.

2: *V, $\leftarrow \cup_{k=1}^{K}V_{k}$* **where** *V*_*k*_ = {node_(*k*-1) min(*m*0, *W*)+1_, node_(*k*-1) min(*m*0, *W*)+2_,…, node _*k* min(*m*0, *W*)_ }

3: **for** *k* **=** 1**:** *K* **do**

4:  *E* ← *E* +{edge _*i*, *j*_} **for all** node_*i*_, node_*j*_ **∈** *V*_*k*_ (*i*≠*j*)

5: **for** *k* **=** 1**:** *K* **do**

6:  *G* = *E + $\cup_{k=1}^{K}V_{k}$*

7: **for** *i* **=** *m*_0_ +1**:** *N* **do**

8:  **add** node_*i*_ **to** *V*

9:  **for** *j* = *m*+1: *N* (*i* ≠ *j*) **do**

10:    **if** deg(node_*j*_)< *W* **and $rank_{node_{j}\in V}\text{random}\left(1\right)\;\text{deg}\left({\text{node}}_{j}\right)\leq m_{0}$**

11:      *E* **←** *E +* {edge _*i*, *j*_}

12:      deg(node_*i*_)← deg(node_*i*_)+1

13:      deg(node_*j*_)← deg(node_*j*_)+1

14:    **end if**

15:  **end for**

16: **end for**

17: **for** *i* **=** *m*_0_+1**:** *N* **do**

18:  *group*_*i*_ ←{}

19:  **for** *j* = *i*+1: *N* (*i* ≠*j*) **do**

20:    **if** *edge*_*i*,*j*_ **∈** *E*

21:      *group*_*i*_ ←*group*_*i*_ U{*node*_*j*_}

22:    **end if**

23:  **end for**

24:  *E* ← *E* +{edge _*k*, *l*_} **for all** node_*k*_, node_*l*_ **∈** *group*_*i*_ (*k≠l*)

25: **end for**

Thus, the two constraints given below are assigned to each node *X* in SFN-SC:

*Constraint 1*. From other nodes, *X* accepts a maximum of *W* edges, including the edges *X* directed to other nodes that arrived in *G* before *X* did, as indicated by *node*_*j*_ ∈ *V* in Line 10. This means that *X* accepts a maximum of *W-m*_0_ neighbors from newer nodes, which arrive in *G* after *X*.

*Constraint 2*. *X* directs an edge only to nodes that joined *G* before *X*.

By combining these two constraints, the degree of each node *X* is bounded by *W*. Specifically, *X* receives edges from newer nodes that arrive after *X* only when deg(*X*) is less than *W*. If *X* directed edges to other *m*_0_ nodes, it can accept additional edges only if *W > m*_0_. Conversely, if *W* ≤ *m*_0_, nodes newer than the *K min*(*W*,*m*_0_),*i*.*e*., *KW*, nodes in the initial clusters generated in Line 2 cannot be connected to those clusters but generate new clusters that grow to have *W* + 1 nodes.

**Fig 2:**
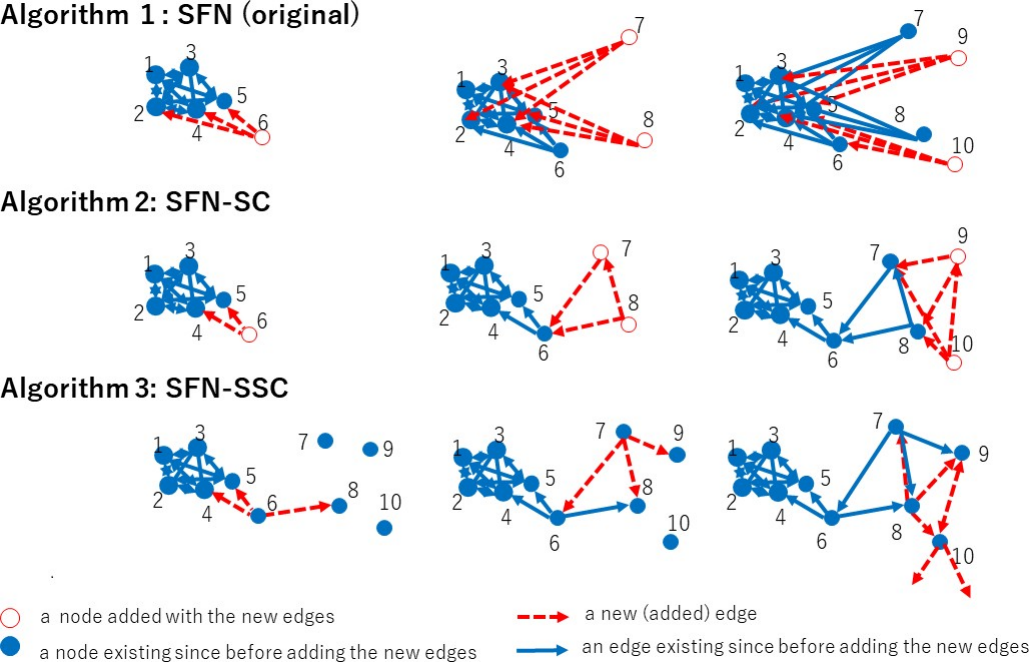
Intuitive comparison of Algorithm 1 (the original SFN in [16]) setting *m*_0_ = 3, Algorithms 2 (SFN-SC) and 3 (SFN-SSC) setting *m*_0_ = 3 and *W* = 4: The red (hollow) nodes and dotted lines indicate new nodes and edges, the blue solid lines were present before the red ones. SFN-SSC makes a wider connection between communities.

However, this does not exactly represent daily-life human experience in real-world urban settings because it assumes that each inhabitant makes spontaneous contact with others only when a new person joins the city, nor that they choose to contact only with those people who were in the city before they had arrived. In this sense, we also chose to consider an alternative procedure in Algorithm 3, where Condition 2 is expired (*node*_*j*_ ∈ *V* in Line 9 no longer means Constraint 2, because *V* includes all the *N* nodes to be included in *G* from the beginning of this algorithm owing to the initial members of *V* specified in Line 1 that differs from Line 2 of Algorithm 2). Owing to this release of the constraints, the degree of each node *X* is not strictly bounded by *W*, which means *X* can choose other nodes to contact even after it has received the edges. See Fig 2 for an intuitive comparison of Algorithm 1, 2, and 3 (setting *m*_0_ = 3, *W* = 4).

**Fig 3:**
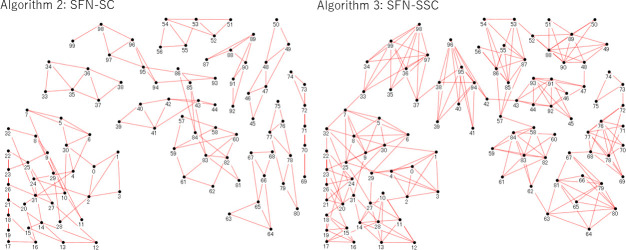
Example of obtained graphs by the two algorithms. SFN-SSC connects clusters although the setting of the number of people each member actually meets (*W*) and the number they choose to meet (*m*_0_) are set equal for the two algorithms.

Algorithm 3 is considered to be “selfish” because a node (node *X*) does not accept edges from other nodes if *X* is too busy for the contacts with *W* other nodes even if *X* itself is partially responsible for becoming busy, which means that the *W* nodes may include nodes to which *X* had directed its edges for its own benefit; however, *X* executes its entitlement to the direct new edges to up to *m*_0_ neighbors even in busy situations. This matches the real-world human society, where a busy person avoids new contacts from others but continues to maintain contact with others to pursue their own business. It is noteworthy that *X*, denoted by node_*i*_ in Algorithm 3, can contact another node_*j*_, where *j* may be larger than *i*, which indicates any node in the entire set *V* that has been filled since Line 1. Owing to the contactabinlity, *X* may endeavor to contact the potential market embracing new contacts that are not yet popular if *X* has room within its edges (*m*_0_) because *X* comes into contact with not only those who chose to contact others before *X* did, but also with those who will do so after it. As shown in Figs 2 and 3, Algorithm 3 expands the connection from a cluster to other nodes (see the connections from node 10). The degree distribution of neither SFN-SC nor SFN-SSC follows the power law, but the most popular nodes take the degree *W* or *W*+*m*_0_. As implied by this difference from the original SFN given by Algorithm 1 (wherein the degree distribution is well known to follow the power-law as mentioned in a previous section), although we borrowed its idea to generate a network from SFN for reasons discussed earlier in the Introduction section, our highlight is not about SFN itself. In this study, we thus highlighted the effect of unintended connections (represented by *W*- *m*_0_) by controlling *W* and *m*_0_.

### Algorithm 3: SFN with selfish spatiotemporal constraints (SFN-SSC)

1: *E* ← {}; *V* ←{node_1_, node_2,…,_node_*N*_}*; G* = *E + V*

2: **for** *k* **=** 1**:** *K* **do**

3:  *V*_*k*_ = {node_(*k*-1) min(*m*0, *W*)+1_, node_(*k*-1) min(*m*0, *W*)+2_, …, node _*k* min(*m*0, *W*)_ }

4:  *E* ← *E* +{edge _*i*, *j*_} **for all** node_*i*_, node_*j*_ **∈** *V*_*k*_ (*i≠j*)

5: **for** *i* **=** *m*_0_+1**:** *N* **do**

6:  deg(node_*i*_)← *m*_0_

7:  **for** *j* = *m*+1: *N* (*i* ≠*j*) **do**

8:    **if** deg(node_*j*_)< *W* **and $rank_{node_{j}\in V}\text{random}\left(1\right)\;\text{deg}\left({\text{node}}_{j}\right)\leq m_{0}$**

9:      *E* **←** *E +* {edge _*i*, *j*_}

10:      **if** deg(node_*j*_) = *W*: *V*←*V*- {node_*j*_}deg(node_*j*_)+1

11:      deg(node_*j*_) ← deg(node_*j*_)+1

12:    **end if**

13:  **end for**

14: **end for**

15: **for** *i* **=** *m*_0_ +1**:** *N* **do**

16:  group_*i*_ ← {}

17:  **for** *j* = *i*+1: *N* (*i* ≠*j*) **do**

18:    **if** edge_*i*,*j*_ **∈** *E*

19:      group_*i*_ ← group_*i*_ U{node_*j*_}

20:    **end if**

21:  **end for**

22:  *E* ← *E* +{edge _*k*, *l*_} **for all** node_*k*_, node_*l*_ ∈ *group*_*i*_ (*k≠l*)

23:  **end for**

### Model of infection spreading dynamics

For each node in the network generated above, three-step dynamics are considered for the spread of infection. The first is to catch a virus from neighboring nodes, such as people, places, or objects in those places, the second is to become infected, and the third is to become an infector to other nodes. Note that each edge in *G* represents the possibility that two entities (people, places, or objects in those places) may come into contact with each other. The activation rate *p*_active_ is defined here as the percentage of “active” edges (generated in Algorithms 2 or 3) connecting the nodes that contact each other in the simulation. In other words, *p*_active_ |*E*| edges are the nodes’ contacts, and all the other edges are shut off. Furthermore, a contact of nodes means that the nodes meet closely enough; for example, a conversation of 15 min at a distance of 2 m, to cause an infection if one of the nodes is infective. If people contact fully and thus activate all edges in *G*, *p*_active_ is 1.0. If the frequency of contacts is reduced by 80% due to governmental regulation, *p*_active_ is set to 0.2. Here, we use the following three notations.

**catch(*i*).** the maximum value of infector(neighbor) of all the contacting neighbors of node_*i*_.

**infected(*i*).** the strength of infection specified by a real value. It is 1 if catch(*i*) is larger than random(0, 1) i.e. a random value ranging with uniform probability between 0 and 1. infected(*i*).

**infector(*i*).** the strength of the node to infect others, equal to 1 with a probability of 0.2 one week after infected(*i*) gets infected i.e. becomes equal to 1. It is set to 0 otherwise. infector(*i*) also fades by *r* per week.

Accordingly, we modeled the dynamics of infection that follows the steps described below week after week.

*Contacting the neighbors*. The rate of *p*_active_ (a real value between 0 and 1) of all the edges in *E* in Algorithm 2 or 3 are randomly chosen. Subsequently, for each node *X*, the other nodes that are connected to *X* are taken as potential contacting neighbors of *X*.

*Catching virus from neighbors*. The *i* -th node gets infected by the strength of catch(*i*) because it catches the virus if any of its contacting neighbors has become an infector. This state does not yet necessarily mean the node is infected. An infector is generated by the following rule:

*Becoming infected*. This stage is concluded when infected(*i*) becomes equal to 1, meaning that the *i*-th node becomes infected with a probability of 0.5 if that node catches the virus from any of its neighbors. Moreover, infected(*i*) is specified here by a real value rather than a discrete judgment considering the uncertainty. The infection of a node fades by a constant *r* (set to 0.7, corresponding to recovery in 4 weeks) each week. This fading, corresponding to recovery in 4 weeks, indicates the deactivation of the virus or the node’s acquisition of immunity.

*Becoming an infector*. If a node is infected, its ability to infect others is indicated by the strength, that is, infectivity infector(*i*). The probability of 0.2 for acquiring infectivity is set here tentatively to the approximate estimation according to announcements such as the one by WHO on April 10, 2020 about the Japanese infection spread.

An infected person is expected to infect others and generate approximately two other infected nodes (see https://rt-live-japan.com/ for the data on effective reproduction numbers) within two weeks. The effective reproduction number 2.0 is larger than the reported number of infection reproductions in the period of spreading [28], but is set to this value to consider the situation in which people interact as usual. And, assuming a probability of 50% that a person becomes infected after catching the virus. Then, he/she infects others with a probability of 0.2. Thus, n * p_active_ * 2 * 0.2 *0.5 should be almost equal to 2.0, where *n* is the number of people each person may meet. Thus, considering *n* = 10, *p*_active_ is regarded as 1.0 in usual daily life interactions with others. Here, 10 is the approximated average number of intimate friends reported for each person in Japan (https://chosa.nifty.com/relation/chosa_report_A20121123/1/).

Infection dynamics have also been modeled by fusing network-based and population-based SIR model [1, 27]. However, we modeled the probabilistic threshold system using the infection process above, where each node *X* becomes infected if catch(*X*) exceeds a threshold given probabilistically. Some findings regarding the recovery of infectiveness after its fading have been reported recently, but we put them out of scope for focusing on the effect of network structures.

## Results

We executed the simulation of 100 weeks/trial by setting the number of nodes (*N*) in *G* to 1,000 and 10,000 and starting in the 0-th week with two infected nodes selected randomly from *G*.

### Tendencies of second waves

**Fig 4:**
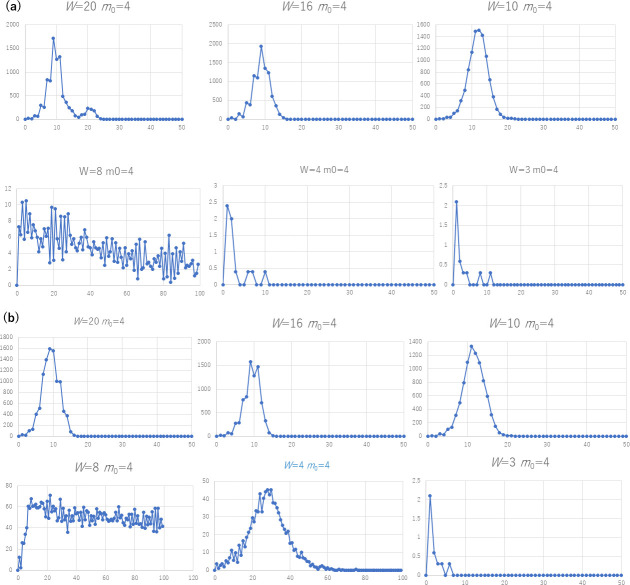
The number of new infection cases (a) *K* = 1 and (b) *K* = 0.1 *N*/*W* of SFN-SC (Algorithm 2, *N* = 10000, *p*_active_ = 1.0) for varying *W* and *m*_0_.

**Fig 5:**
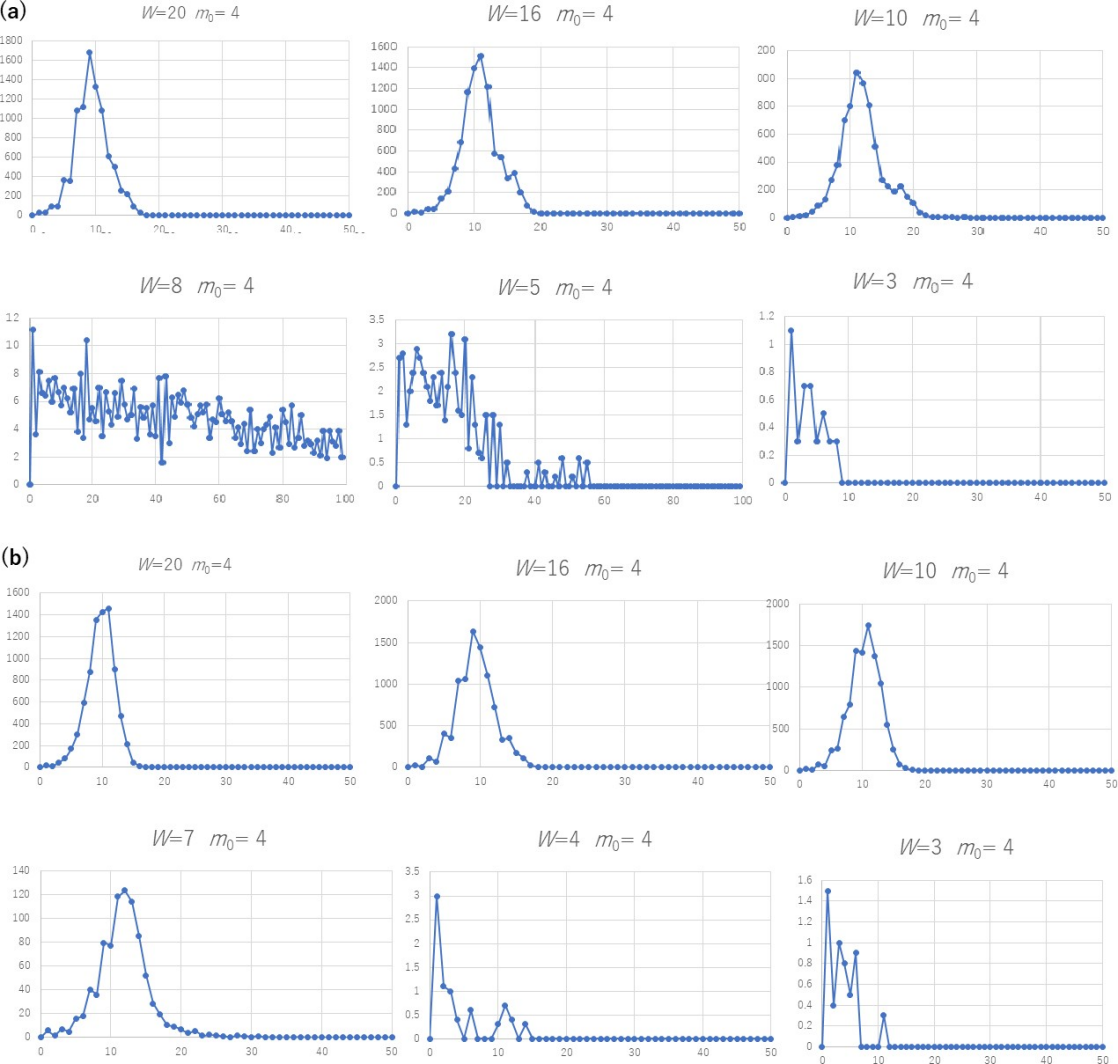
The number of new infection cases (a) *K* = 1 and (b) *K* = 0.1 *N*/*W* of SFN-SSC (Algorithm 3, *N* = 10000, *p*_active_ = 1.0) for varying *W* and *m*_0_.

Figs 4 and 5 plot the average number of new infection cases per week, using Algorithms 2 and 3 for 10 trials. Note that each curve does not show a history obtained in one trial, but a mixture of the sequences of the averaged trials. The number of initial clusters is set to *K* = 0.1 *N*/*W* in order to create separate clusters for a large *W*. For example, if the number of nodes (*N*) is 10,000 and *W* is 20, we start with 50 cliques as the initial clusters. Figs 4 and 5 indicate several tendencies. First, the peaks of the second wave, if any, tend to be lower than the peaks of the first wave in cases where the network structure do not change; for example the first and the fifth subfigure in Fig 4(A), the third in Fig 5(A), etc. as far as the structure of the social network remains invariant in the time series.

Here, let us define a peak by the time *t* where the number of new infection cases (*t*) is equal to $\underset{t-\Delta t\leq s\leq t+\Delta t}{\max}cases\left(s\right)$ with Δ*t* set to 5 weeks. We detected the second peak in the curves of 45.7% of all (729 among 1600) tested cases. Specifically, we considered twenty values of *W* from 1 to 20, five values of *m*_0_ (1, 2, 4, 6, and 8), two types of initial cliques (single and multiple), two values of *N* (1,000 and 10,000), for the two algorithms, for two constant activation rates *p*_active_ (0.5 and 1.0) throughout the 100 weeks of time. The second peak was higher than the first one for 10% (160 cases) of all cases. The third peak was higher than the first in 3.7% (59 cases). In 1.2% (19 cases), the curve showed a second peak after the number of new infection cases had decreased to less than 5% of the first peak, where the first peak had 10 or more infected cases.

**Fig 6:**
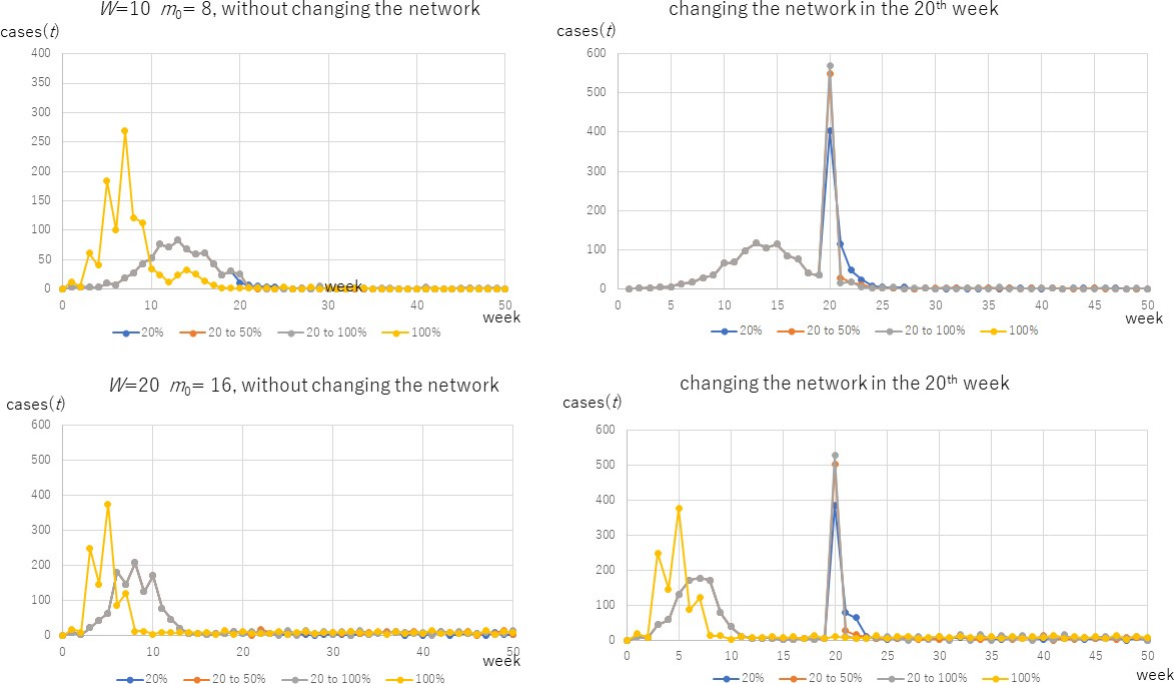
The effect of changing the network structure: The effect of Scenario C in Fig 1.

Next, we changed the network at the 20^th^ week from one generated from multiple cliques to one from a single initial clique without changing *N*, *W*, *m*_0_, or the infection status (infected(*X*), infector(*X*), catch(*X*) defined in the model of the infection) of any node. Here, we tested only for Algorithm 3 because our aim is to investigate the changes in the inter-cluster structure that is mode dominant in an SFN-SSC than in an SFN-SC, as shown in Fig 2. For this reason, and also because the SFN-SSC reflects the above-mentioned “selfish” nature of people in contacting others, we considered the results of Algorithm 3, i.e., SFN-SSC. That is, as illustrated in Scenario C in Fig 1, the structure of the entire graph *G* is changed here. Consequently, as shown in the second peaks in Fig 6, we obtained the second finding: we detected a second peak in 58.3% (350cases) among the 600 tested cases. Specifically, we considered twenty values of *W* (integers from 1 through 20), five values of *m*_0_ (1, 2, 4, 6, 8), three values of *p*_active_ (0.2, 0.5, and 1.0), and two values of *N* (1,000 and 10,000). In 35% (213 of all cases), the second peak was higher than the first peak. Furthermore, 22.2% (133 cases) of all the curves exhibited a second peak following the structural change, after it had decreased to less than 5% of the first peak.

### Tendencies of infections for varying *W* and *m*_0_

**Fig 7:**
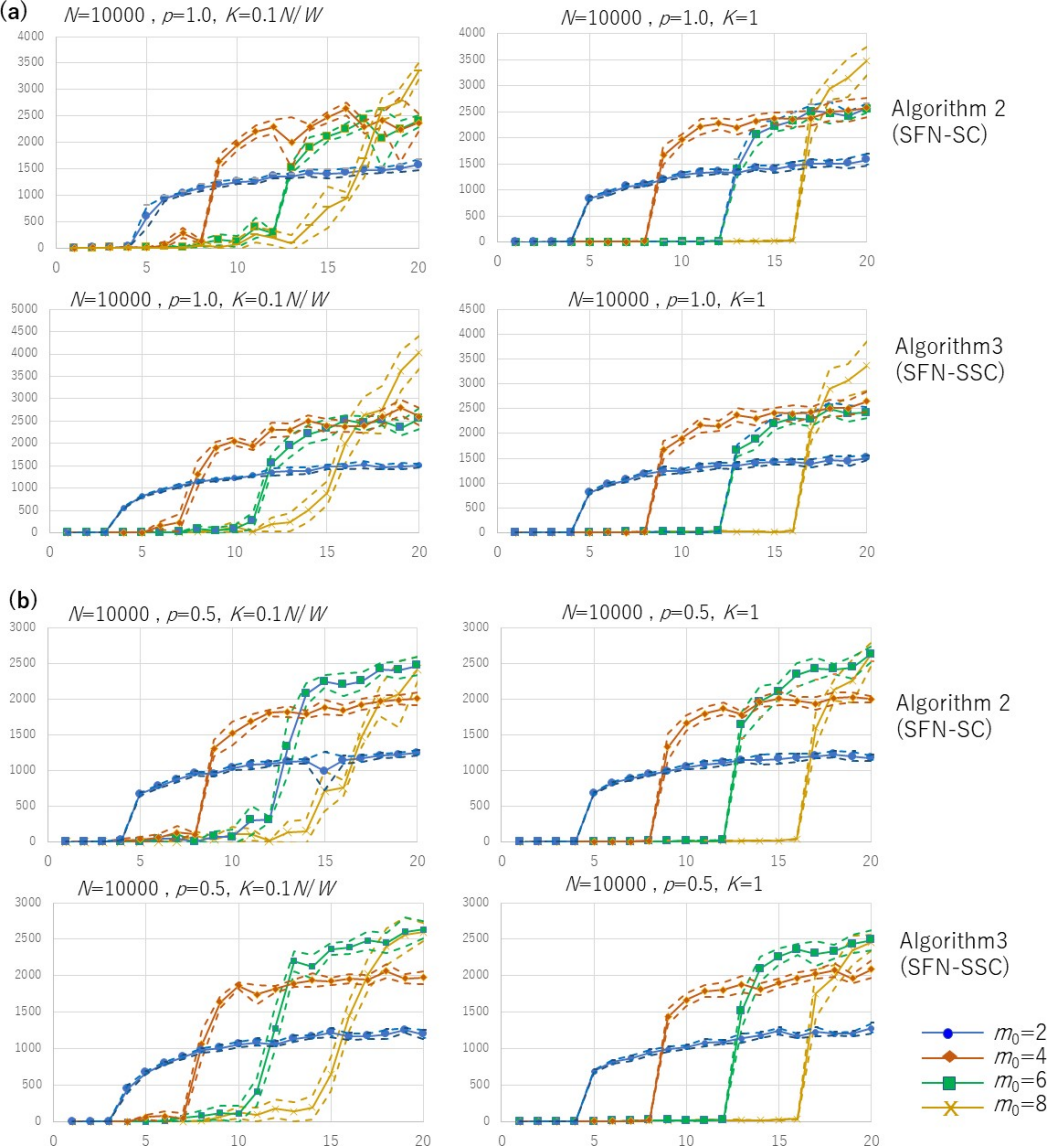
The maximum number of new infection cases in *Y* axis, for *K* = 1 and *K* = 0.1 *N*/*W* of SFN-SC and SFN-SSC, *N* = 10000, with varying *W* and *m*_0,_ for (a) *p*_active_ = 1.0 and (b) *p*_active_ = 0.5. The dotted lines indicate the confidence interval of 95%.

The third tendency we observe from the curves that are exemplified in Figs 4 and 5 is that the peak of the number of infection cases tends to vary for different values of *W* and *m*_0_. In the S1 to S16 Tables in S1 File, we recognize two. First, the number of infection cases positively depends on *W* according to the values in the cells in each column. Second, a larger value of *m*_0_ does not always result in a larger number of new infection cases. For example, in the rows where *W* is between 9 and 16, we found that the largest number of infections tend to occur for 2 ≤ *m*_0_ ≤ 6 rather than for a larger *m*_0_. Rather, we observed a substantial increase when the value of *W* increased from *W* < *m*_0_ to *W* ≥ 2*m*_0_. This tendency is graphed in Fig 7. For the cases of multiple initial cliques (*K* = 0.1 *N*/*W*), two-step growth, that is, the subtle increase when *W* goes over (i.e., from smaller to larger than) *m*_0_ and the noticeable increase when *W* goes over 2*m*_0_. The increase at *W* of 2*m*_0_ is especially noticeable for the case of a single initial clique (*K* = 1). As shown by the dotted lines showing the upper and lower bounds of the 95% confidence interval, the increase at *W* = 2*m*_0_ is significant.

**Fig 8:**
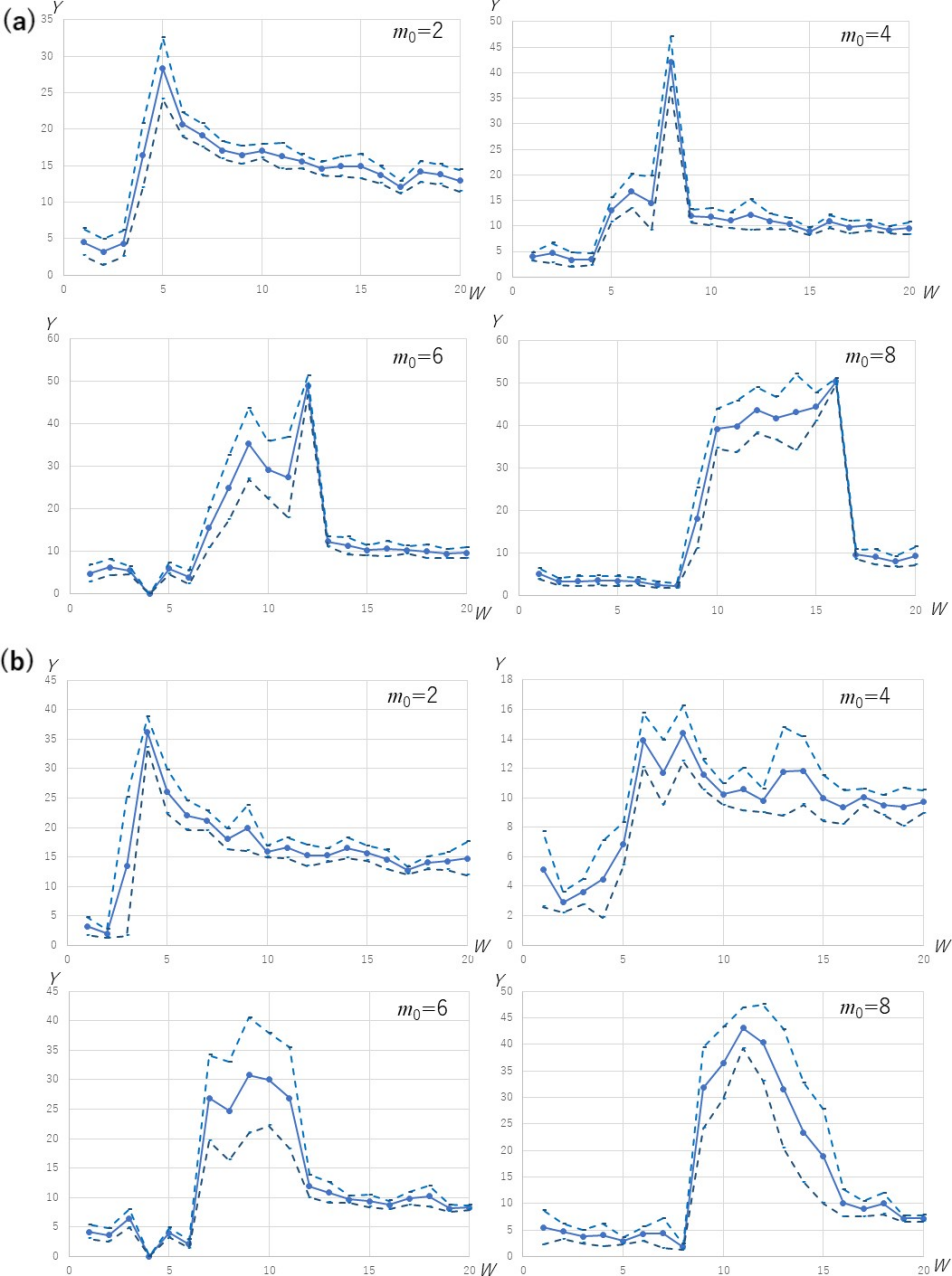
The average week of infections in *Y* axis, for *N* = 10000 and *p*_active_ = 1.0 of SFN-SSC with varying *W* and *m*_0_ for (a) *K* = 1 and (b) *K* = 0.1 *N*/*W*. The dotted lines indicate the confidence interval of 95%.

When *W* exceeds *m*_0_, although the increase in the number of new infections is not significant, the average peaks start to shift to a later week, as shown in Fig 8. The values in the *Y* axis represent the expected timing of infections, with the first infections in this network set as the 0-th week. The value of *Y* here increases discontinuously with the increase in *W* from *m*_0_+1 until it exceeds 2*m*_0_. Here, we restricted the results to SFN-SSC (Algorithm 3) for *p*_active_ of 1.0 and *N* = 10000, and other results are shown in the right-hand sides of S1 to S16 Tables in S1 File where a similar tendency can be observed in Fig 8A and 8B. Moreover, in these tables, a third tendency is observed: the number of infection cases per week tends to rise radically for *W* > 2*m*_0_, and the delay of peaks occurs for *W* > *m*_0_. The reasons behind these tendencies are discussed in the Discussion section.

### Effects of releasing restrictions

**Fig 9:**
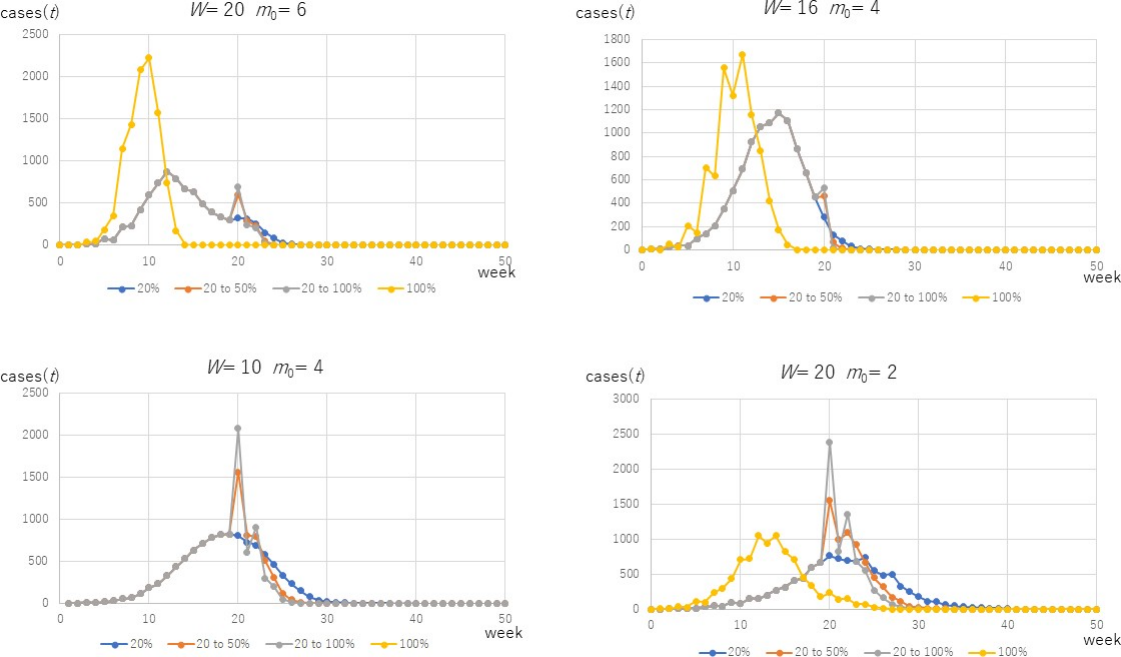
The number of new infection cases before and after releasing the restriction for SFN-SSC, *N* = 10000.

Fig 9 graphs the examples of releasing restrictions of *p*_active_ = 0.2 to 0.5, and eventually, to 1.0, which indicates completely free contact and communication with the neighbors in *G*. In this example, the release at a time close to the peak causes a drastic new peak even higher than the peak in the case of free communication (*p*_active_ = 1.0), whereas the release in the down-going slope at a later time does not.

**Fig 10:**
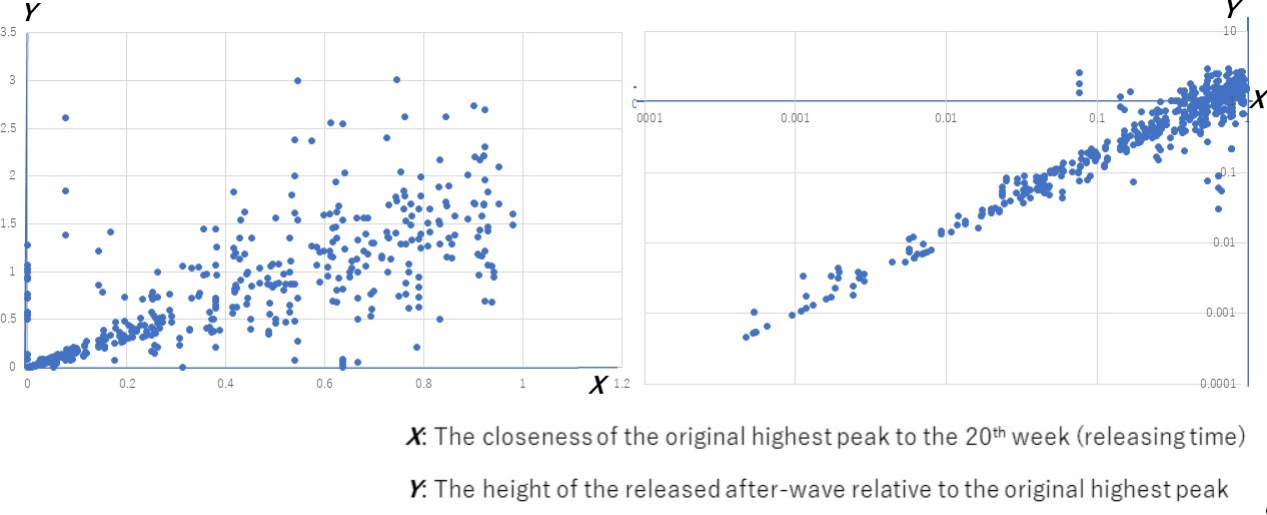
The impact of releasing the restriction early. *X*: the closeness of the original (i.e., *p* = 0.2) highest peak to the 20^th^ week and *Y*: the height of the released (i.e., *p* = 1.0) second wave relative to the original highest peak.

We then executed the simulations by changing the parameters to validate this tendency. Here, the restrictions were released in the 20^th^ week in all the 2000 tested cases that were simulated. This includes five independent cycles under 400 conditions. Specifically, we considered twenty values of *W* (integers from 1 through 20), five values of *m*_0_ (1, 2, 4, 6, 8), only multiple initial cliques were considered, two values of *N* (1,000 and 10,000), and two *p*_active_ values (0.5 and 1.0), for which the restriction has been released from *p*_active_ of 0.2. Among these 2000 cases, we found 762 cases where the first peak of the 20% was before the 20^th^ week and cases(*t*) were 10 or larger at the peak. We evaluated the correlation of *X*: the closeness of the original (i.e., *p*_active_ = 0.2) highest peak to the 20^th^ week and *Y*: the height of the released (i.e., *p*_active_ = 1.0) second wave relative to the original highest peak. *X* and *Y* in Fig 10 are defined as follows:

$$X:={\;\text{Max}}_{0.2,\;20\leq t\leq 100}\;\text{cases}\left(t\right)/{\text{Max}}_{0.2,\;0\leq t\leq 100}\;\text{cases}\left(t\right)$$ (2)

$$Y\;:={\text{Max}}_{1.0,\;20\leq t\leq 100}\;\text{cases}\left(t\right)/{\text{Max}}_{0.2,\;0\leq t\leq 100}\;\text{cases}\left(t\right)$$ (3)

Here, cases(*t*) define the number of new infection cases in the *t*-th week. In Eq (2), Max_0.2, 0≤*t* ≤100_ cases(*t*) and Max_0.2, 20≤*t*≤100_ cases(*t*) are the largest number of cases(*t*) for all the simulated weeks and for the period after the release in the 20^th^ week, respectively, with *p*_active_ = 0.2 for both. The larger *X* indicates the closeness of the 20^th^ week to the highest peak before the 20^th^ week because *X* is reduced if the 20^th^ week appears after the peak. Note that *X* is not the sheer temporal distance; instead it is the relative delay considering the decrease in the number of infection cases, thereby reflecting our aim to investigate the influence of time on reducing cases(*t*). In Eq (3), Max_1.0, 20≤*t*≤100_ cases(*t*) denotes the largest number of cases(*t*) after the release in the 20^th^ week to *p*_active_ = 1.0. A larger *Y* indicates a stronger impact of the release because *Y* approaches 1.0 from a smaller value if the value of Max_1.0, 20≤*t*≤100_ cases(*t*), which means the largest number of infection cases after the release, is equal to or larger than the largest number before the release.

As shown in Fig 10, a significant correlation (Pearson’s correlation = 83.0, *p <* 0.0001) between *X* and *Y* was obtained for the 762 cases as mentioned earlier. Therefore, these result indicate that the sooner the release is after the peak in a restricted period, the higher the wave caused after the release.

## Discussion

According to the simulation results, the first finding is that the peak of the second wave, if any, tends to be lower than that of the first wave assuming an invariant network structure. This tendency can be explained with the help of Scenario A presented in Fig 1. As shown, the infection cases monotonously fade after the peak. On the other hand, in Scenario B, the second wave occurs in a localized cluster in the network. Unless the localized clusters are connected, the infection in one cluster, if any, fades without an interactive activation with other clusters. This first finding can be explained by the dynamics of a network with distributed local clusters bridged through weak ties.

The second finding from the simulation results is that the second peak higher than the first occurs more often if the network changes its own structure either in the period infection spreads or after the infection has been suppressed. This is because the infectors directly touch non-infected people who have not acquired immunity. This implies a risk of an outbreak if people start inter-community interactions, even at a time when the observed infection spread has settled.

The third finding is that the number of infection cases positively depends on *W* according to S1 to S16 Tables in S1 File, the largest allowed number of people that a person actually meets. However, the dependency on *m*_0_ is not always positive. For example, in the rows where *W* ranges between 9 and 16, we found that the largest number of infections occurred for 2 ≤ *m*_0_ ≤ 6 rather than for a larger *m*_0_. From each table, we find that this is a result of the tendency mentioned above that the peak of the number of infection cases increases drastically with the increase in *W* over 2*m*_0_. Therefore, we cannot always simply propose people to keep a smaller number of neighbors. The risk is reduced significantly for *m*_0_ = 1, that is, having only one neighbor to meet because this would decrease the probability of casting and catching of the virus. However, restricting *m*_0_ to 1 is too strong for an ordinary way of life. Therefore, we propose a realistic method for suppressing infection spreading, which reduces the number of participants in a group (i.e., *W*-*m*_0_). The results of Fig 7 and S1 Table in S1 File show that reducing *W*-*m*_0_ to 0 contributes to suppressing the number of infected cases. The reason for this is clear: if *W* is equal to *m*_0_ or smaller, a new node cannot be connected to a cluster where all the nodes have a degree of *m*_0_ or larger in Algorithms 2 and 3. In such a case, no cluster can increase in size, and the spread is therefore restricted to each small cluster. However, restricting *W*-*m*_0_ to 0 means accepting no meeting with nodes in external clusters, which is another difficult constraint for businesses where it is required to create contracts with new collaborators or shops that need to accept visits from new customers. Furthermore, the effect of this restriction of *W* from larger than *m*_0_ to less than it does not prove as effective as the restriction of *W* from larger than 2*m*_0_ to a value less than it, in the sense that *W =* 2*m*_0_ is the more significant bound of the increase in the number of new infections.

On the other hand, because the clusters of nodes come to be connected via narrow paths when *W* is larger than *m*_0_ by a small value, the infection comes to spread slowly to a wider range in the network because of this connection. That is, the clusters start to bridge in the range of *m*_0_ ≤ *W* < 2*m*_0_, which enables infections through long paths in *G*, thus taking a longer time than in each small cluster. For a larger *W*, the paths are widened owing to the added edges, the infections are accelerated, and consequently, the time required for spreading is reduced. Thus, the value of *Y* in the result of Fig 8 increases discontinuously with the increase in *W* from *m*_0_+1, until it exceeds 2*m*_0_.

Accordingly, to suppress the peaks, which is a strategy to avoid the overload of hospitals and other social facilities, we propose to pay attention to the second threshold *W* = 2*m*_0_, as shown in Fig 7, for the two types of networks generated from both single and multiple initial cliques. The condition *W* > 2*m*_0_ causes radically high peaks, which can be explained by considering the two-step phenomenon that is caused as follows:

Step 1) A node *X* is added to *G* with *m*_0_ edges from *X* to the existing nodes. As a result, *m*_0_ as part of the capacity of *G*, which can accept connections from new nodes via new edges, is lost.

Step 2) *X* itself has the capacity to accept *W- m*_0_ edges, which are then added to the capacity of *G*. Therefore, if W–*m*_0_ ≥ *m*_0,_ i.e., if *W* ≥ 2*m*_0_, each cluster in *G* obtains more capacity by adding new nodes. Otherwise, the growth of each cluster stops when it reaches a limited size because each cluster in *G* loses its capacity to acquire new connections.

Based on this, we propose that each node, for example, a person, does not actually meet others that they do not choose (maintain *W* < *m*_0_) and, if this is too strict, a person should not accept unintended contacts from a as large number of people as one chooses (maintain *W*–*m*_0_ < *m*_0_). To clarify, *W*–*m*_0_ denotes the number of other people that a person actually meets unintentionally. This proposal means that a person should remain in the community that was chosen intentionally rather than meeting others from external communities. This concept is in agreement with the findings in the literature [1, 29] that mixing heterogeneous people or connecting nodes via a long-distance edge can expand the infection. The effect of a bridge between communities is just one interpretation of *W*–*m*_0_ and *W*– 2*m*_0_ under the assumption that directing edges to other nodes of *m*_0_ is an intentional action from a node.

Finally, the fourth tendency we found is that the release of a previously imposed constraint may trigger a second wave higher than the peak of the curve for *p*_active_ = 1.0, which indicates the fastest spread when no constraints enforced, if the release is introduced soon after the peak. Thus, the restriction, if introduced once, should not be released quickly without checking the results of the step-wise release. For example, the government should release the constraint when *p*_active_ = 0.2 to 0.3, while still restricting access to public places where people from different communities often meet, then monitor the effect on the number of infection cases, and only proceed to 0.4 if a substantial increase does not occur.

The results of this study can be associated with the assertion that a risk of infection spreading in a society exists where people in each household decide to maintain an in-person social connection with one person from another household [30]. In comparison, the policies we suggested above are less strict but realistic and practical proposals are required for the decision-making of both individuals and policymakers. Furthermore, by combining a stochastic model for state transitions and networked multi-agent simulations, Cremonini and Maghool [31] showed that undetected (asymptomatic or mildly ill) cases may still exist and cause complex social influence even after the peak of the pandemic wave. In our study, the asymptomatic cases corresponded to nodes infected but were not chosen probabilistically to be infectors before they lost infectivity. These nodes are surrounded less densely by infected nodes than by infectors because infectors can infect their neighbors. In this sense, the risk here is relevant to the case of second waves in Scenario B. Karsai et al. [32] found that curbing of the spread is caused by weight-topology correlations. The weight here refers to the frequency of communications via each edge, whereas the topology corresponds to the location of each edge, depending on whether it is in a cluster or a bridge. By interpreting that frequent communication across bridges between communities accelerates the spread of infection, we can verify similar tendencies in a different setting. An algorithm for reconstructing the strong ties that are the invariant backbone structure of a network, distinguished from weak ties constructed from transient links in the dynamic network structure, from data on the individuals’ status in the spreading process [33], has been shown to be useful for a targeted immunization strategy that prioritizes influential nodes in the inferred backbone. Our conclusion may differ from this result in that we showed that bridges between clusters contributed to the spreading, which seems to correspond to weak ties rather than to strong ties. However, these weak ties called bridges between clusters that we considered herein are not necessarily as transient as the weak ties defined by Surano et al. in [33] and are particularly influential to various clusters, which is consistent with their findings.

## Conclusion

The spread of infection over human networks was simulated where the constraints were specified by the maximum number of people that each person actually meets (*W*) and the number of people that they choose to meet (*m*_0_). The real-space daily life of people in urban settings was modeled as a modified SFN with constraints on the values of the two abovementioned factors *W* and *m*_0_. Consequently, the following results were obtained: to suppress the number of infections, we require policies that focus on the bridges between densely infected clusters rather than considering only the clusters. We recommend that the government should not release previously introduced restrictions on social contact too soon, and careful attention must be paid to the results of step-by-step release of constraints. Likewise, individuals should avoid contact with people apart from the ones that they choose to meet during the period of infection spread. This means that people should remain in their intimate community with people they have chosen to be with. Even if this constraint is too strict, one should not accept additional contacts from a larger number of people as compared to those that one had chosen to maintain contact with during the period of infection spread. The reader should not misunderstand that one can meet as many people as they like provided that the number of additional contacts is less than that of the chosen contacts. If one enjoys such freedom, the person who becomes contacted may touch more than 2*m*_0_ others if it is not an intended contact for both sides. Instead, one should establish a community with those they had chosen to maintain contact with during the period of infection spread and then prevent any new contacts following the abovementioned rule.

In this and future studies, we are approaching stepwise, confirming that each step is supported by simulations and on real data as evidence. For example, in this study, following the assumption of the original SFN, *m*_0_ was considered constant (same value) for all members of the society, which were represented by nodes in the network, as required in the established original way of generating an SFN. Similarly, *W* was set equal to all members for studying its relationship with *m*_0_. Our next step is to consider the variety of *W* and for different nodes to fit the real society and apply it to real world data on the living of various people in real cities, whereas the present paper aims to show just one step of extension from SFN to considering the spatiotemporal constraints.

In a similar sense, we intentionally restricted the dynamics of the network to a one-time change to clarify the effect of each change. This is realistic for the release of *p*_active_ to a larger value because the release of a governmental restriction usually occurs in stages in the case of a country or an administrative district. However, in our future steps we shall consider the continuous changes in the network structure considering that a real society undergoes continuous changes when the members of the public are allowed to freely interact with each other. Therefore, our new target of research on temporal networks is to enable the extended analysis of network epidemiology by referring to real data [34] that is obtained by collecting essential datasets using methods for finding and creating useful data [35].

## Supporting information

S1 File (XLSX)
